# Molecular Epidemiology and MLST-Based Typing of Pandrug-Resistant *Acinetobacter baumannii* Clinical Isolates in Iraq: A Cross-Sectional Study

**DOI:** 10.30699/ijp.2025.2052426.3412

**Published:** 2025-03-10

**Authors:** Hani Hasan Jubair, Marwa Jabbar Mezher, Noor Ayyed Mayea

**Affiliations:** 1 *Department of Pathological Analysis, Faculty of Science, University of Kufa, Najaf, Iraq*; 2 *Department of Medical Biotechsnology, College of Biotechnology, Al-Qadisiyah University, Diwaniyah, Iraq*; 3 *Department of Optometry Techniques, Technical Institute of Al-Najaf, Al-Furat Al-Awsat Technical University, Najaf, Iraq*

**Keywords:** Acinetobacter baumannii, pandrug-resistant, Multilocus Sequence Typing, OXA-23

## Abstract

**Background & Objective:**

*Acinetobacter baumannii* is a globally recognized nosocomial pathogen capable of developing multidrug resistance. This study investigates antibiotic resistance patterns, evaluates common resistance genotypes, and explores the genetic relatedness of PDR *A. baumannii* clinical isolates from hospitals in the Middle Euphrates region of Iraq.

**Methods:**

Fourteen PDR *A. baumannii* isolates were obtained and subjected to antimicrobial susceptibility testing using the Vitek-2 compact system. Resistance genes were identified via conventional PCR, and clonal relationships were analyzed using multilocus sequence typing (MLST).

**Results:**

Among 175 *A. baumannii* isolates, 8% (14/175) were classified as PDR strains, exhibiting resistance to all tested antibiotics. TEM was the most prevalent resistance gene (50%), followed by CTX-M (43%). SHV, IMP, KPC, OXA-48, and Mcr-1 genes were absent in all PDR isolates. MLST analysis identified five sequence types (STs): ST2, ST218, ST138, ST123, and ST460, with ST2 being the most common (50%).

**Conclusion:**

The high prevalence of PDR *A. baumannii strains* in Iraq highlights the need for enhanced antibiotic surveillance. A comprehensive molecular investigation is necessary to mitigate the spread of these resistant pathogens.

## Introduction

*Acinetobacter baumannii* is a Gram-negative bacterium responsible for nosocomial infections and has recently increased in prevalence. This opportunistic pathogen is well-known for its ability to develop resistance to multiple antibiotics, posing a significant challenge for healthcare professionals (1). The emergence of multidrug-resistant (MDR) and extensively drug-resistant (XDR) strains of *A. baumannii* is a major concern for public health and clinical practice due to their increased resistance patterns, which diminish treatment effectiveness (2). Recent studies indicate a rise in XDR *A. baumannii* strains in Middle Eastern countries (3,4).

The emergence of PDR *A. baumannii* strains has raised significant concerns within healthcare environments. These bacteria demonstrate resistance to all known antibiotics, leading to the development of pan-drug resistance (5). This phenomenon is facilitated by mechanisms such as efflux pumps and genetic mutations. Understanding the genetic determinants of PDR is crucial for developing effective strategies to combat these highly drug-resistant organisms (6).

The high detection frequency of antibiotic resistance genes in *A. baumannii* isolates represents a significant public health risk. The genetic relatedness of *A. baumannii* to hospital environments and its association with multidrug resistance episodes highlight substantial genetic variation in resistance mechanisms (7). This variation includes multi-class β-lactamases and carbapenemases in clinical isolates (4,8). Intensive surveillance and a targeted treatment approach are required to control this bacterium. Epidemiological trends must be closely monitored to improve infection prevention and treatment strategies against *A. baumannii* isolates (9).

Molecular methods such as MLST, which employ multiple loci in the bacterial genome, allow for the determination of isolate lineages and their clonal relationships. This technique characterizes the genetic and evolutionary differences of PDR strains of *A. baumannii* (10). Research has shown that MLST profiles ST1 and ST2 are associated with specific resistance genes, such as OXA-23, providing insights into the genetic basis of resistance within clonal complexes (11). The study of MLST diversity in relation to resistance mechanisms offers a better understanding of *A. baumannii's* adaptability to antibiotic exposure, underscoring the importance of continuous monitoring and molecular analysis in the fight against multidrug-resistant organisms (9,10).

The emergence and spread of PDR *A. baumannii* in Middle Euphrates hospitals remain poorly understood. Limited studies have characterized its resistance genes and molecular epidemiology, particularly through MLST. This study aims to investigate the dissemination of PDR strains, identify resistance genes, and analyze the correlation between sequence types (STs) and antibiotic resistance patterns, providing insights into the mechanisms driving antibiotic resistance in *A. baumannii*.

## Materials and Methods

### Sample Collection and Bacterial Identification

A cross-sectional study was conducted on PDR *A. baumannii* isolates collected from patients attending ten hospitals in the Middle Euphrates region between January 2023 and April 2024. A total of 175 clinical samples, including blood, urine, sputum, wound discharge, cerebrospinal fluid (CSF), and bronchoalveolar lavage (BAL), were collected prior to antibiotic administration. The University of Kufa (registration number 102) granted this study's ethical approval. Clinical information, including age and sex, was anonymized to maintain patient privacy. Samples were stored in Amies Charcoal medium at room temperature and processed within two hours. Specimens were cultured on MacConkey, Blood, and Chrom agars at 44°C in a 5% CO2 incubator. *A. baumannii* was confirmed through standard microbiological assays and the Vitek-2 bacterial identification system.

### Antibiotic Susceptibility Testing

Antimicrobial susceptibility was evaluated using the Vitek-2 compact system with the AST-GN222 card, following Clinical and Laboratory Standards Institute (CLSI) 2023 guidelines (12). The MDR, XDR, and PDR resistance phenotypes were classified based on established cut-off values (13).

### Molecular Detection of Antibiotic Resistance Genes

#### DNA Extraction

According to the manufacturer's instructions, DNA extraction from PDR A. baumannii isolates was performed using a GeneJET Purification Kit (Thermo Scientific, USA). Extracted DNA samples were stored at -20°C for subsequent PCR analysis.

#### PCR Amplification

PCR amplification of specific targets in *A. baumannii* isolates was performed using different primer sets and DNA extracted from one isolate. Table 1 provides details of the antibiotic resistance genes used in this study, along with amplification conditions and annealing temperatures.

The PCR reaction was conducted in a 25 μL volume containing 12.5 μL of 2× Taq PCR MasterMix (Beijing ComWin Biotech Co. Ltd., Beijing, China), 9.5 μL of ddH₂O, 1 μL of forward primer, 1 μL of reverse primer, and 1 μL of extracted template DNA. The amplification conditions were as follows: initial denaturation at 94°C for 5 min, followed by 31 cycles of 94°C for 30 s, annealing at various temperatures depending on the target gene (as shown in Table 1) for 45 s, and extension at 72°C for 30 s to 1 min. A final extension step was performed at 72°C for 10 min.

PCR products were verified by electrophoresis on a 1.5% agarose gel. The bands were visualized using an ultraviolet (UV) transilluminator (UV Tech, France) after staining with DL2000 DNA Marker (TaKaRa, Dalian, China).

#### Multilocus Sequence Typing (MLST)

MLST analysis was performed on 14 PDR *A. baumannii* clinical isolates based on PCR amplification of seven housekeeping genes: *cpn60, fusA, gltA, pyrG, recA, rplB,* and *rpoB*. The PCR reaction was conducted in a 50 μL final volume, with the following cycling conditions: initial denaturation at 94°C for 2 min, followed by 35 cycles of 94°C for 30 s, annealing at 50°C for 30 s, and extension at 72°C for 30 s. A final extension step at 72°C for 5 min was performed before cooling the samples to 4°C.

Sangon Biotech Company sequenced PCR products. The allelic profiles for each housekeeping gene were combined to determine sequence types (STs). ST assignment was conducted using the MLST online database (https://pubmlst.org/bigsdb?db=pubmlst_abaumannii_seqdef&page=profiles).

### Statistical Analysis

Data were analyzed using IBM SPSS (SPPS Inc, Chicago, IL, USA). Significant variables were assessed using the chi-square test. Descriptive statistics were presented using relative frequency distributions. A *p*-value of ≤0.05 was considered statistically significant

**Table 1:** The gene primers utilized for the PCR amplification of antibiotic resistance genes in PDR clinical isolates *A.baumannii*

	Gene name	Oligo sequence (5'-3')	Product size (bp)	Annealing temperature	Reference
ESBLs	TEM	F: TCGCCGCATACACTATTCTCAGAATGA	445	45
		R: ACGCTCACCGGCTCCAGATTTA		
	SHV	F: ATGCGTTATATTCGCCTGT	747	55
		R: TGCTTTGTTATTCGGGCCAA		
	CTX	F: TCTTCCAGAATAAGGAATCCC	554	60
		R: CCGTTTCCGCTATTACAAAC		
	CTX-M	F: ATGTGCAGTACCAGTAAGCGTCATGGC	593	52
		R: TGGGTAAAATATGTCACCAGAACCAG		
oxacillinases	OXA-23	F: GATCGGATTGGAGAACCAGA	501	52
		R: ATTCTGACCGCATTTCCAT		
	OXA-24	F: GGTTAGTTGGCCCCCTTAAA	246	52
		R: AGTTGAGCGAAAAGGGGATT		
	OXA-51	F: TAATGCTTTGATCGGCCTTG	353	52	
		R: TGGATTGCACTTCATCTTGG			
	OXA-48	F: GCGTGGTTAAGGATGAACAC	438	52
		R: CATCAAGTTCAACCCAACCG		
Carbapenemase	VIM	F: GATGGTGTTTGGTCGCATA	390	65
		R: CGAATGCGCAGCACCAG		
	IMP	F: GGAATAGAGTGGCTTAAYTC	232	60
		R: TCGGTTTAAYAAAACAACCACC		
	KPC	F: CGTCTAGTTCTGCTGTCTTG	789	58
		R: CTTGTCATCCTTGTTAGGCG		
	NDM	F: GGTTTGGCGATCTGGTTTTC	621	65
		R: CGGAATGGCTCATCACGATC		
colistin	MCR-1	F: AGTCCGTTTGTTCTTGTGGC	320	58	
		R: AGATCCTTGGTCTCGGCTTG			

## Results

This study analyzed 175 non-duplicative *A. baumannii* isolates collected from various clinical specimens, including wound discharge (68, 38.8%), urine (51, 29%), blood (26, 14.8%), sputum (13, 7.4%), cerebrospinal fluid (CSF) (11, 6.2%), and bronchoalveolar lavage (BAL) (6, 3.4%). The isolates were sourced from different hospital wards: burns centers (78, 44.5%), urology (45, 25.7%), infectious diseases (25, 14.3%), surgery (17, 9.7%), and intensive care units (ICU) (10, 5.7%).

### Antimicrobial Susceptibility Testing

**Fig. 1:**
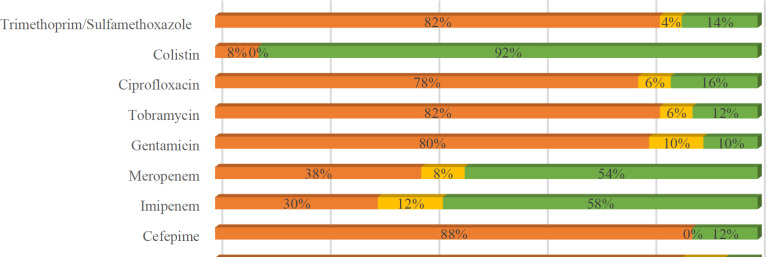
Antimicrobial susceptibility testing of 175 *A. baumannii* clinical isolates.

Antimicrobial susceptibility testing of the 175 *A. baumannii* isolates revealed a high prevalence of antibiotic resistance. The results are illustrated in Figure 1. Most isolates exhibited resistance to cephalosporins (ceftazidime and cefepime), aminoglycosides (gentamicin and tobramycin), and β-lactamase inhibitors (ticarcillin, ticarcillin/clavulanic acid, piperacillin, and piperacillin/tazobactam). Furthermore, the majority of *A. baumannii* isolates were resistant to trimethoprim/sulfamethoxazole and ciprofloxacin. In contrast, carbapenems (imipenem and meropenem) had the lowest resistance rates. Although colistin, a lipopeptide antibiotic, was the most effective agent, 14 isolates (8%) displayed resistance.

According to the results of antibiotic susceptibility testing and the criteria proposed by Magiorakos et al. (13), multidrug-resistant (MDR) isolates were defined as those resistant to at least one antibiotic in three or more antimicrobial classes. Among the 175 *A. baumannii* isolates, 129 (73.3%) were classified as MDR, while 32 (18.2%) were considered extensively drug-resistant (XDR). Notably, only 8% (14/175) of isolates were resistant to all 13 antibiotics tested, classifying them as pandrug-resistant (PDR) agents.

### Molecular Characterization of Resistance Genes

Fourteen *A. baumannii* isolates, phenotypically confirmed as PDR, were subjected to PCR experiments to detect extended-spectrum β-lactamases (ESBLs), carbapenemase, oxacillinase, and colistin resistance genes. The PCR results revealed heterogeneous distribution of antibiotic resistance genes among the 14 PDR isolates, with some isolates harboring multiple resistance genes.

PCR analysis for ESBL genes showed that TEM, CTX-M, and CTX were detected in seven (50%), six (43%), and one (7%) of the PDR *A. baumannii* isolates, respectively (Table 2). The SHV gene was not found in any PDR *A. baumannii* isolates.

Carbapenemase genes were also investigated. VIM and NDM genes were present in three (21%) and one (7%) isolates, respectively, while no amplification was detected for IMP and KPC genes.

Regarding oxacillinase-encoding genes, five (36%) isolates harbored the OXA-23 gene, three (21%) had the OXA-51 gene, and two (14%) carried the OXA-24 gene. None of the isolates contained the OXA-48 gene. Additionally, none of the PDR *A. baumannii* isolates possessed the *mcr-1* colistin resistance gene.

### Multilocus Sequence Typing (MLST) Analysis

MLST analysis was performed to evaluate the genetic diversity of the PDR *A. baumannii* isolates. The 14 isolates were classified into five distinct sequence types (STs), based on the "Oxford" MLST scheme (Table 3). ST2 and ST218 were the most prevalent, accounting for seven (50%) and three (21%) isolates, respectively. Other sequence types identified included ST138 (n = 2), ST123 (n = 1), and ST460 (n = 1).

**Table 2:** Distribution of antibiotics resistance genes among PDR *A. baumannii* isolates (n=14)

Isolate		Antibiotic resistance genes							
		TEM	CTX-M	CTX	VIM	NDM	OXA-23	OXA-24	OXA-51
PDR-AB1		+	+	-	-	-	+	-	-
PDR-AB2		-	-	-	+	-	-	+	-
PDR-AB3		+	-	+	-	-	-	-	-
PDR-AB4		+	+	-	-	+	+	-	-
PDR-AB5		-	-	-	-	-	-	-	+
PDR-AB6		-	+	-	+	-	-	-	-
PDR-AB7		+	-	-	-	-	-	+	-
PDR-AB8		-	-	-	-	-	-	-	-
PDR-AB9		-	+	-	-	-	+	-	-
PDR-AB10		+	-	-	-	-	-	-	-
PDR-AB11		-	-	-	-	-	-	-	+
PDR-AB12		+	+	-	+	-	-	-	-
PDR-AB13		-	-	-	-	-	+	-	-
PDR-AB14		+	+	-	-	-	+	-	+
Total	**No.**	**7**	**6**	**1**	**3**	**1**	**5**	**2**	**3**
	**%**	**50%**	**43%**	**7%**	**21%**	**7%**	**36%**	**14%**	**21%**

(-) denotes negative, (+) denotes positive PCR reaction.

**Table 3:** Sequence types (STs), type of samples, and wards of 14 PDR A. baumannii isolates in the Middle Euphrates region.

**Isolate**	**Sequenced housekeeping genes (Allelic profile)**							**(STs)**	**Sample**	**ward**
	*gltA*	*gyrB*	*gdhB*	*recA*	*cpn60*	*gpi*	*rpoD*			
PDR-AB1	**2**	**2**	**2**	**1**	**1**	**2**	**7**	**2**	**Wound**	**Burns centers**
PDR-AB2	**1**	**3**	**3**	**2**	**2**	**50**	**3**	**138**	**Wound**	**Burns centers**
PDR-AB3	**2**	**2**	**2**	**1**	**1**	**2**	**7**	**2**	**Wound**	**Burns centers**
PDR-AB4	**2**	**2**	**2**	**1**	**1**	**2**	**7**	**2**	**Wound**	**Burns centers**
PDR-AB5	**4**	**12**	**4**	**11**	**4**	**100**	**5**	**123**	**Wound**	**Burns centers**
PDR-AB6	**1**	**3**	**3**	**2**	**2**	**102**	**3**	**218**	**Blood**	**ICU**
PDR-AB7	**2**	**2**	**2**	**1**	**1**	**2**	**7**	**2**	**Wound**	**Burns centers**
PDR-AB8	**1**	**3**	**3**	**2**	**2**	**102**	**3**	**218**	**Wound**	**Burns centers**
PDR-AB9	**2**	**2**	**2**	**1**	**1**	**2**	**7**	**2**	**Wound**	**Burns centers**
PDR-AB10	**2**	**2**	**2**	**1**	**1**	**2**	**7**	**2**	**Wound**	**Burns centers**
PDR-AB11	**65**	**17**	**107**	**11**	**1**	**156**	**6**	**460**	**Urine**	**Surgery**
PDR-AB12	**1**	**3**	**3**	**2**	**2**	**102**	**3**	**218**	**Wound**	**Burns centers**
PDR-AB13	**1**	**3**	**3**	**2**	**2**	**50**	**3**	**138**	**Blood**	**Infectious diseases**
PDR-AB14	**2**	**2**	**2**	**1**	**1**	**2**	**7**	**2**	**Wound**	**Burns centers**

## Discussion

*Acinetobacter baumannii* has emerged as an opportunistic pathogen with increasing isolation rates in hospitals, particularly within intensive care units and burn centers. The rising resistance to all antibiotics presents a significant public health concern. This bacterium's remarkable ability to acquire multidrug resistance complicates treatment regimens and contributes to high morbidity and mortality rates in infected patients (17,18).

According to the antimicrobial resistance patterns observed, 38% of strains were resistant to meropenem, 30% to imipenem, and 8% to colistin. Other antibiotic classes, including cephalosporins, aminoglycosides, and β-lactamase inhibitors, also exhibited high resistance rates. The colistin resistance observed in this study was comparable to findings from India, China, and Lebanon, which reported resistance rates of 8.2%, 11.8%, and 17.5%, respectively (19). The increasing prevalence of multidrug-resistant strains is alarming, as *A. baumannii* demonstrates resistance to critical antibiotics, including colistin, meropenem, and imipenem (8,20).

Most previous studies have focused on MDR and XDR *A. baumannii* isolates. This study is the first to examine the dissemination of PDR *A. baumannii* strains in Iraq, revealing antibiotic resistance gene profiles with similar MLST patterns. A total of 14 (8%) PDR *A. baumannii* isolates were obtained from hospitals within the same district, suggesting possible transmission between these isolates. This is crucial for epidemiology and antibiotic resistance surveillance, as *A. baumannii* is classified as a priority pathogen. A similar study in Egypt reported a PDR prevalence of 2.2% (21), while in Oman, the prevalence was 1% (22). The increasing occurrence of PDR *A. baumannii* poses a critical challenge in healthcare settings, highlighting the selective pressure exerted by carbapenems and ciprofloxacin, which, along with clonal expansion, promotes the spread of resistant strains.

Molecular analysis of PDR *A. baumannii* isolates is essential for understanding their genetic profiles and resistance patterns. Previous studies have used PCR to analyze antimicrobial susceptibility and resistance genes to guide interventions and surveillance efforts (21,23). In this study, PDR *A. baumannii* strains were predominantly ESBL-positive, with 50% carrying the TEM gene, 43% harboring the CTX-M gene, and 7% carrying the CTX gene. Al-Sheboul et al. reported a high prevalence of PDR *A. baumannii* in Jordanian ICUs, with most strains being ESBL-positive and harboring TEM and CTX-M genes (24).

The identification of carbapenemase genes was another critical finding. Three isolates carried VIM genes, while one harbored the NDM gene. These genes are associated with the high prevalence of PDR strains and limited treatment options. Studies in Indonesia identified carbapenem-resistant *A. baumannii* with a high prevalence of carbapenemase production, where the VIM gene was predominant (25). National monitoring of carbapenem-resistant *A. baumannii* isolates is essential to prevent their spread. A study in Egypt revealed that carbapenem resistance is driven by multiple β-lactamase enzymes, including Class A, B, C, and D β-lactamases (21).

Additionally, oxacillinase-encoding genes were found to be prevalent, with OXA-23, OXA-51, and OXA-24 detected in 36%, 21%, and 14% of isolates, respectively. This suggests that oxacillinase enzymes play a crucial role in antibiotic resistance in PDR *A. baumannii* strains. Investigations in eastern China highlighted the emergence of epidemic PDR *A. baumannii* strains carrying the OXA-23 gene (26). Further molecular analysis of PDR *A. baumannii* isolates revealed that the most frequently detected genes were OXA-23 and OXA-51, with some isolates also carrying OXA-24, further complicating the resistance profile of *A. baumannii* (27). The findings underscore the need for enhanced surveillance and control programs to prevent the spread of PDR *A. baumannii* strains. These studies also emphasize the significance of genotyping in epidemiological analysis and infection management (1,11).

Molecular epidemiology plays a crucial role in understanding *A. baumannii* strain relatedness and antibiotic resistance lineages. Given these challenges, molecular typing methods like MLST are indispensable. MLST characterizes bacterial strains by sequencing conserved housekeeping genes, allowing for detailed genetic analysis of *A. baumannii* populations and inter-isolate relationships (28). Our findings identified five sequence types (ST2, ST218, ST138, ST123, and ST460), with ST2 being the most prevalent, representing 50% of PDR *A. baumannii* strains from hospitals in the Middle Euphrates region. This study provides the first MLST analysis of PDR *A. baumannii* isolates in Iraq. These sequence types may indicate intercontinental transmission, as they have been identified in isolates from multiple countries. Previous studies have linked certain sequence types to resistance profiles, suggesting that specific genetic backgrounds may increase the likelihood of pan-drug resistance (11).

The predominance of ST2 among PDR *A. baumannii* isolates, especially in burn centers, underscores the importance of local epidemiological surveys to control their dissemination. A study by Hojabri et al. conducted MLST analysis on *A. baumannii* strains from an Iranian hospital, demonstrating the predominance of ST2 strains associated with severe infections, increased multidrug resistance, and elevated mortality rates (29). Similarly, Liu et al. in China used MLST to characterize carbapenem-resistant *A. baumannii* isolates, identifying diverse sequence types and common carbapenemase gene combinations (30). The high resistance levels observed in PDR *A. baumannii* isolates, particularly to carbapenems, underscore the global threat posed by these pathogens. Our study demonstrated the genetic diversity of isolates, with ST2 being the most prevalent among clinical samples in the Middle Euphrates region. These findings align with global reports on the widespread dissemination of specific sequence types (31,32).

Integrating MLST data into antibiotic susceptibility testing can enhance the accuracy of treatment outcome predictions and optimize clinical decision-making. Therefore, utilizing MLST to predict antibiotic response in *A. baumannii* infections offers significant promise for improving patient care and addressing the global challenge of antibiotic resistance.

## Conclusion

This study highlights the high prevalence of pandrug-resistant (*A. baumannii*) strains in clinical isolates from hospitals in the Middle Euphrates region of Iraq, emphasizing the urgent need for enhanced surveillance and antibiotic resistance studies. Detecting multiple resistance genes, including *NDM, VIM, TEM, OXA-23,* and *OXA-51*, underscores the necessity of comprehensive molecular epidemiology studies to mitigate the spread of PDR strains.

Many PDR *A. baumannii* isolates exhibited resistance to carbapenems and colistin, further complicating treatment options. The application of multilocus sequence typing (MLST) as a molecular typing method has provided valuable insights into the genetic variation and evolutionary relationships of *A. baumannii* strains. Understanding the epidemiology and transmission of these drug-resistant isolates through MLST is critical for developing effective infection control strategies.

Overall, these findings emphasize the importance of molecular surveillance and genomic characterization in addressing the rising threat of PDR *A. baumannii*. The integration of MLST and molecular epidemiology can aid in monitoring the dissemination of resistant strains and inform targeted interventions to combat antibiotic resistance. 

## Ethical Approval

This study received ethical approval from the University of Kufa (registration number 102) for collecting samples from bacteriological laboratories in various hospitals within the Middle Euphrates region. 

## Funding/Support

This research did not receive any specific grant from public, commercial, or not-for-profit funding agencies.

## Data Reproducibility

Data are available upon reasonable request from the corresponding author. 
